# Sex-limited diversification of the eye in *Heliconius cydno* butterflies

**DOI:** 10.1007/s00359-025-01768-z

**Published:** 2025-10-16

**Authors:** Nathan P. Buerkle, Nicholas W. VanKuren, Erica L. Westerman, Marcus R. Kronforst, Stephanie E. Palmer

**Affiliations:** 1 Department of Organismal Biology and Anatomy, University of Chicago, Chicago, USA; 2 Department of Ecology and Evolution, University of Chicago, Chicago, USA; 3 Department of Biological Sciences, University of Arkansas, Fayetteville, USA; 4 Department of Physics, University of Chicago, Chicago, USA; 5 Present Address: Department of Neuroscience, Yale University, New Haven, CT USA

**Keywords:** Butterflies, Color vision, Photoreceptors, Sexual dimorphism

## Abstract

Butterflies have evolved a remarkable diversity in eye organization to support a range of vision-based behaviors including courtship, oviposition, and foraging. This diversity has been surveyed extensively across the butterfly phylogeny, but variation across closely related species remains less clear. We compared eye organization in *Heliconius cydno*, a clade of mimetic, Neotropical butterflies that have been studied in the context of wing coloration and courtship. Using a combination of eyeshine and opsin immunohistochemistry, we identified several sexually dimorphic features of eye organization where male eyes varied with species and female eyes did not. These features included the distribution of a red screening pigment across the eye, co-expression of the two UV opsins within single photoreceptors, and the relative distribution of UV and blue opsin expression in R1/R2 photoreceptors. Together, this suggests a shift in *H. cydno* males from an ancestor strongly biased towards the expanded Nymphalid mosaic characterized by blue and long wavelength opsin co-expression, red screening pigment, and green vs. red inter-photoreceptor opponency to one biased towards the basic mosaic consisting of UV-UV, Blue-Blue, and UV-Blue ommatidia. We hypothesize that this sex-limited variability may function to adapt these butterflies to sexually dimorphic behaviors like courtship and oviposition in the context of the natural light environment.

## Introduction

The organization of peripheral sensory systems plays an important role in behavior by specifying what environmental information is available to an animal. This organization appears to be evolutionarily labile and subject to potentially rapid adaptations that often function to increase the detectability of behaviorally relevant stimuli or more broadly tune the system to natural scene statistics (Osorio and Vorobyev 2008; Bendesky and Bargmann 2011). In the visual system, photoreceptor spectral sensitivities can evolve through several means including opsin gene duplication and divergence, co-expression of multiple opsins in the same cell, or the use of filtering pigments that influence which wavelengths reach a photoreceptor (Briscoe and Chittka 2001; Stavenga and Wilts 2014). For example, in an adaptive radiation of African cichlids there are seven opsin genes, with differences in expression patterns across the eye that adapt an animal to ambient light conditions at different depths as well as sexually selected conspecific color patterns (Torres-Dowdall et al. 2017). Further highlighting the evolvability of the visual periphery, mantis shrimps have at least 16 photoreceptor types constructed from more than 30 opsin genes (Cronin et al. 2014), while dragonflies can express more than 30 unique long-wavelength sensitive opsins (Futahashi et al. 2015).

**Fig. 1:**
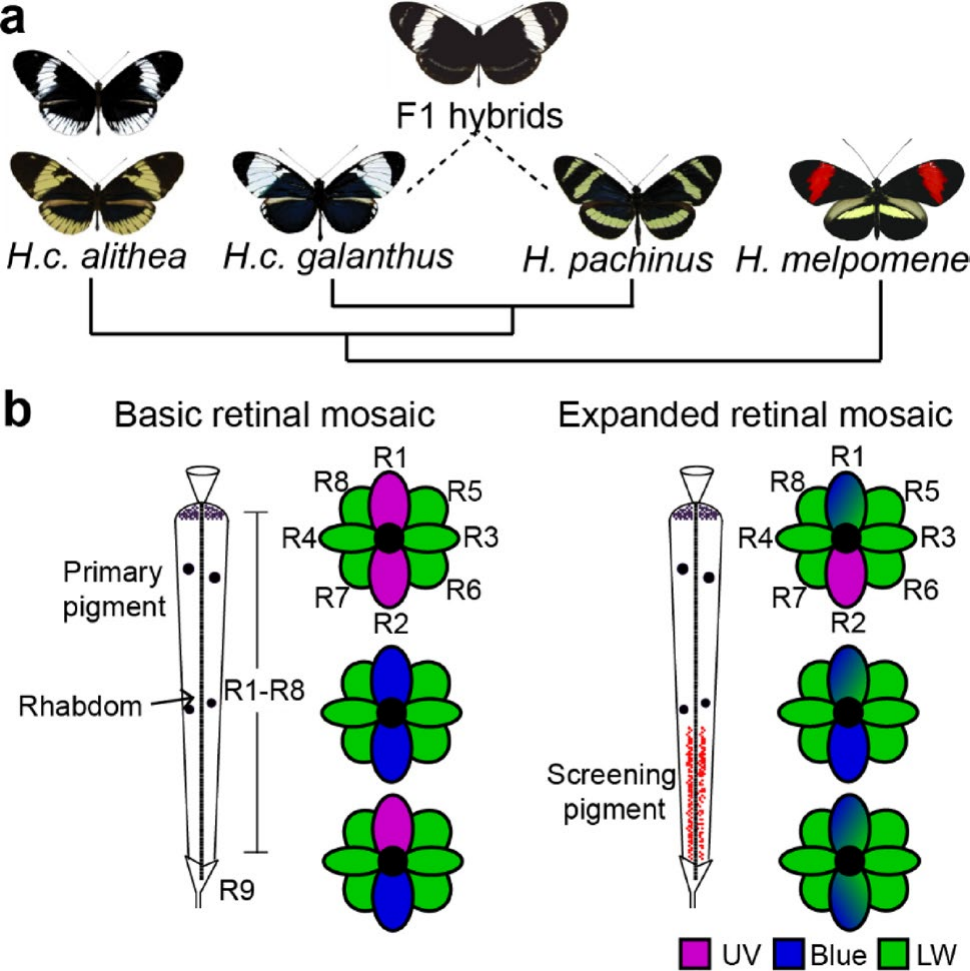
Study system **a **Phylogenetic tree showing the species studied here, including the hybrid offspring of two sister species. **b** The Nymphalid eye comprises ommatidia in the basic retinal mosaic, matching the likely ancestor of all butterflies and the expanded retinal mosaic, characterized by red screening pigment, co-expression of the LW and B opsin in R1/R2 and distinct photoreceptor connectivity

Butterflies are another group where the visual periphery has evolved an immense diversity in eye organization from an ancestral eye that likely comprised ultraviolet (UV), blue (B), and long wavelength (LW) sensitive photoreceptors (Briscoe 2008). Some butterflies retain this trichromatic arrangement, with three ommatidial classes that tile the retina. These ommatidial classes are defined by opsin expression in R1 and R2 cells (UV-UV, B-B, and UV-B) (Fig. 1), while R3-8 typically express the LW opsin. Elaborations on this basic organization are common and often sexually dimorphic (Arikawa et al. 2005; Sison-Mangus et al. 2006; Ogawa et al. 2012; Ilić et al. 2022; Pirih et al. 2022), with different screening pigments and duplications of each opsin documented. Red-sensitive photoreceptors are relatively common (Qiu and Arikawa 2003; Arikawa 2003; Zaccardi et al. 2006; Frentiu et al. 2007; Arikawa et al. 2009; Blackiston et al. 2011), and the number of photoreceptors with unique spectral tuning varies across taxa. The well-studied *Papilio xuthus* has eight photoreceptor types (Arikawa 2003), while the *Graphium sarpedon* with 15 has the most reported among butterflies (Chen et al. 2016).

Eye diversity has been examined extensively across the butterfly phylogeny, but it is less clear how this organization varies among closely related species. Different features of eye organization could be more or less amenable to adaptive change, and contrasting phylogeny-wide comparisons with those of closely related groups could help distinguish which aspects are more easily altered. Thus, we focused on characterizing eye organization in a group of closely related *Heliconius cydno* butterflies (Fig. 1). For Nymphalid butterflies like *Heliconius*, eyes exhibit the basic retinal mosaic including UV-UV, B-B, and UV-B ommatidia. Some species, and potentially the ancestral Nymphalid, additionally have an expanded retinal mosaic characterized by a subset of ommatidia with red screening pigment, LW opsin expression in at least one of the R1/R2 cells, and distinct photoreceptor connectivity (Ilić et al. 2022; Pirih et al. 2022). In *Heliconius* and some other Nymphalids, these LW photoreceptors co-express the B opsin (McCulloch et al. 2022). *Heliconius* have an additional layer of retinal complexity due to the duplication of the UV opsin (UV1: λ_max_ = 355 nm and UV2: λ_max_ = 390 nm) (Briscoe et al. 2010). A genus-wide comparison of UV opsin expression patterns has revealed at least six unique retinal mosaics that are typically sexually dimorphic (McCulloch et al. 2017).

For the *H. cydno* butterflies we examined, white vs. yellow wing color is a Mendelian trait important for both mimicry and courtship (Kronforst et al. 2006; Chamberlain et al. 2009; Westerman et al. 2018). Homozygous males preferentially court females of the same wing color, while heterozygotes court both colors equally. We recently showed sex-limited diversity in UV photoreceptor physiology where male eyes varied both in spectral sensitivity and in inter-photoreceptor inhibition that correlated with male preferences (VanKuren et al. 2025). Using a combination of eyeshine and opsin immunohistochemistry, we extend these results to further characterize the organization of the *H. cydno* eye. We consistently observed sex-limited variability in the distribution of red screening pigment in the eye, the distribution of each ommatidial class, and which UV opsin is expressed. Together, these results suggest that *H. cydno* males have substantially shifted eye organization towards the basic mosaic compared to females and the closely related *H. melpomene* without major changes to the relative distribution of blue and UV opsins.

## Methods

### Animals

The butterflies used in this study were housed in a greenhouse at the University of Chicago that was regularly supplemented with new butterflies from breeders located in Ecuador (*H. c. alithea*) and Costa Rica (*H. c. galanthus* and *H. melpomene*). *H. pachinus* and F1 hybrid offspring of *H. c. galanthus* and *H. pachinus* crosses were reared in Panama and transported to the University of Chicago for experiments. All butterflies were at least 3 days old at the time of experiments.

### Eyeshine

Eyeshine images were collected using a custom built epi-fluorescent microscope following a published design (Stavenga 2002). Collimated white light (DH-2000 S, Ocean Optics) was first expanded through a telescope (f = 40 and 80 mm, Edmund optics) and focused through a half-silvered mirror onto the butterfly eye through a 20X, 0.4 NA objective (Zeiss LD-Plan-Neofluar). The resulting eyeshine image was magnified using 80 and 20 mm lenses placed confocally with each other and photographed with a digital camera equipped with an infinity focused lens (Canon EOS Rebel T5). To capture an image, butterflies were restrained in a custom made collar using beeswax and placed on a rotating platform near the focal point of the imaging lens and the position was adjusted using three linear actuators. The butterfly was dark adapted for at least one minute before each image. After an image, the butterfly was rotated to a new, non-overlapping position along the dorsal-ventral axis of the eye.

We quantified the eyeshine distribution by counting the number of red and yellow ommatidia across the entire dorsal-ventral axis of the eye. For each butterfly we took 10–12 images that amounted to 3,686.9 ± 758.2 ommatidia and ~ 30% of the entire eye (Seymoure et al. 2015). Ommatidia were counted in each image blind to species, sex, eye location, and individual. A randomly selected 20% of the images were included twice to ensure repeatability, finding 1–2% differences in the proportion and total number of ommatidia counted. We report proportions based on the two most dorsal images and the ventral half. Results were qualitatively and quantitatively similar when different numbers of images were combined.

### Eyeshine spectral reflectance

We measured the spectral reflectance of individual red and yellow ommatidia using monochromatic light. We began with a reference photo with white light to classify each ommatidia as red or yellow. We then rerouted the white light through a monochromator (MonoScan-2000, Ocean Optics) and replaced the digital color camera with a monochromatic camera (Prosilica GX1050, Allied Vision Technologies). To ensure consistent illumination across stimuli, we measured the photon flux at each wavelength and normalized it relative to the mean flux across the spectral range. We empirically determined that a photon flux of 1.0 × 10^15^ photons produced monochromatic eyeshine images that filled the dynamic range of the camera. We then adjusted the shutter time for each wavelength to achieve this photon flux (6.6 ± 1.1 s). We validated this method by capturing images using a mirror in place of a butterfly eye. Pixel intensities were similar across all wavelengths, indicating that photon flux was similar and wavelength specific changes in camera sensitivity were negligible. Since white light stimuli quickly induce pigment migration and adaption of the eyeshine, we also performed control experiments by recording reflectance spectra before and after an extended exposure (15 minute) to low intensity monochromatic light, which showed measurements were stable and not affected by the longer shutter times used for this experiment.

For each butterfly, we measured reflectance spectra in the dorsal, middle, and ventral part of the eye. These were measured from 500 to 800 nm in 10 nm steps, with preliminary experiments showing no reflectance outside this range. Individual ommatidia were analyzed with ImageJ (Schindelin et al. 2012). We imported the series of monochromatic images as a z-stack, which allowed us to manually select the same ROI for each ommatidium and wavelength. The reflectance at each wavelength for each ommatidium was then defined as the average pixel intensity in the ROI. The images collected were 8-bit for intensity values and we excluded ommatidia from further analysis if the maximum intensity was less than 50% or greater than 95% of the cameras dynamic range.

### Immunohistochemistry

We stained thin cross-sections of the eye with antibodies against UV1, UV2, and blue opsins. The anti-blue opsin antibody was generated against the peptide INHPRYRAELQKRLPC in rabbits and was provided by Michael Perry (Perry et al. 2016). We created our own antibodies specific to UV1 and UV2 proteins. For UV1, the antibody was generated in guinea pigs against the peptide GLDSADLAVVPEC. For UV2, the antibody was generated in mouse against the peptide GLSSAELEFIPEC. Peptide synthesis, conjugation, immunizations, and affinity purification were performed by GenScript (USA).

Eyes were dissected in 0.01 M phosphate buffered saline (PBS) and then fixed at room temperature for 15 min in 4% paraformaldehyde in PBS. Eyes were washed 3 × 10 min in PBS and then cryoprotected overnight at 4 °C in 25% sucrose in PBS before sectioning at 14 μm on a cryostat. With the exception of some eyes that examined the most dorsal part of the eye, we targeted the ventral part of the middle eye for all experiments. After drying overnight, slides were first washed in chilled acetone for 5 min, 2 × 10 min in PBS, 2 × 10 min in 0.3% TritonX-100 in PBS (PBST), 1 × 5 min in 1% sodium dodecyl sulfate in PBST, and 3 × 10 min in PBST. Slides were then blocked for one hour in 1% bovine serum albumin in PBST. Primary antibody was applied overnight at 4 °C as 1:300 dilutions in blocking solution. The following day, slides were washed 5 × 10 min in PBST before applying the secondary antibody. Secondary antibodies (Abcam) were diluted 1:2000 in blocking solution and applied to the slides for 2 h at room temperature. These antibodies were goat anti-rabbit Alexafluor 488, donkey anti-guinea pig Alexafluor 555, and donkey anti-mouse Alexafluor 647. After staining, slides were finally washed 5 × 10 min in PBST and stored in Polymount (Fisher Scientific). Eye slices were imaged using a Zeiss LSM 510 confocal microscope using a 20X objective.

We quantified the distribution of ommatidial classes by counting the number of UV-UV, B-B, and UV-B ommatidia in each butterfly using an automated program. We first generated a binary mask for each antibody as well as one for the merged image. Ommatidia were automatically segmented and identified using the MATLAB function *bwareafilt* on the binary mask of the merged image. We overlayed these segmented ommatidia boundaries on the single channel masks and defined an ommatidium as opsin positive if at least 4% of the ommatidium was filled. This threshold best matched previously reported results for *H. melpomene* (McCulloch et al. 2017) and ensured that presumably LW expressing cells with low levels of co-expression were detected. We validated this program in two ways. First, we manually counted ommatidia for 8 butterflies, blind to the results of the automated program, and found an average difference of 4.1 ± 1.3% in the proportion of blue photoreceptors detected. For each eye, we also ran the program on 2–4 consecutive sections, each of which contained the same ommatidia. Proportions were averaged together across sections, and if the proportion of blue photoreceptors differed by more than 5% the individual was excluded from further analysis. The resulting distributions were analyzed using hierarchical clustering based on the Euclidean distance between the three ommatidial classes measured and using an average linkage function.

### qPCR

We measured the relative difference in UV1 and UV2 mRNA expression using qPCR. Eyes were dissected from the butterfly and immediately placed in RNA-later and stored at −80°C. Before RNA extraction, eyes were washed 5 × 5 minutes in PBS. RNA was extracted and converted to cDNA using a Qiagen RT-PCR kit. UV1 primers were 5’-CGCTCACTGTGTGCTTCCTCTT-3’ and 5’-AGTCTTGCAAGCTACCGCGG-3’. UV2 primers were 5’-TACCGTGTGCTTCCTTTATGTTG-3’ and 5’-ACCCTTGCAAGCGATCGCAG-3’. We also tested for the LW opsin and elongation factor 1 alpha as controls for the quality of RNA extraction. Expression levels were measured in triplicate for each sample using iTaq SYBR Green Supermix (Bio-Rad, USA) and a CFX96 qPCR machine (Bio-Rad, USA), and results with standard deviations greater than 1 across the replicates were discarded and re-tested.

### Comparing eyeshine and antibody staining

We used a simple least squares approach to relate the proportion of red and yellow eyeshine to the proportion of UV-UV, B-B, and UV-B ommatidia we observed with antibody staining. This approach enabled us to estimate the proportion of blue expressing photoreceptors that co-express the LW opsin since all B photoreceptors in ommatidia with red eyeshine should co-express. For each group, we fit the following equation to our data:

$$ Yellow~eyeshine = UVUV + \left( {1 - p} \right)^{2} \cdot BB + \left( {1 - p} \right) \cdot UVB $$

where p is the probability that a blue cell co-expresses LW and is associated with the expanded mosaic and red screening pigment. (1-p)^2^ gives the probability that neither blue cell expresses LW, and (1-p) gives the probability that the only B cell does not co-express. Since the same butterflies were not always used for both eyeshine and antibody staining, we fit this equation using group averages.

**Fig. 2:**
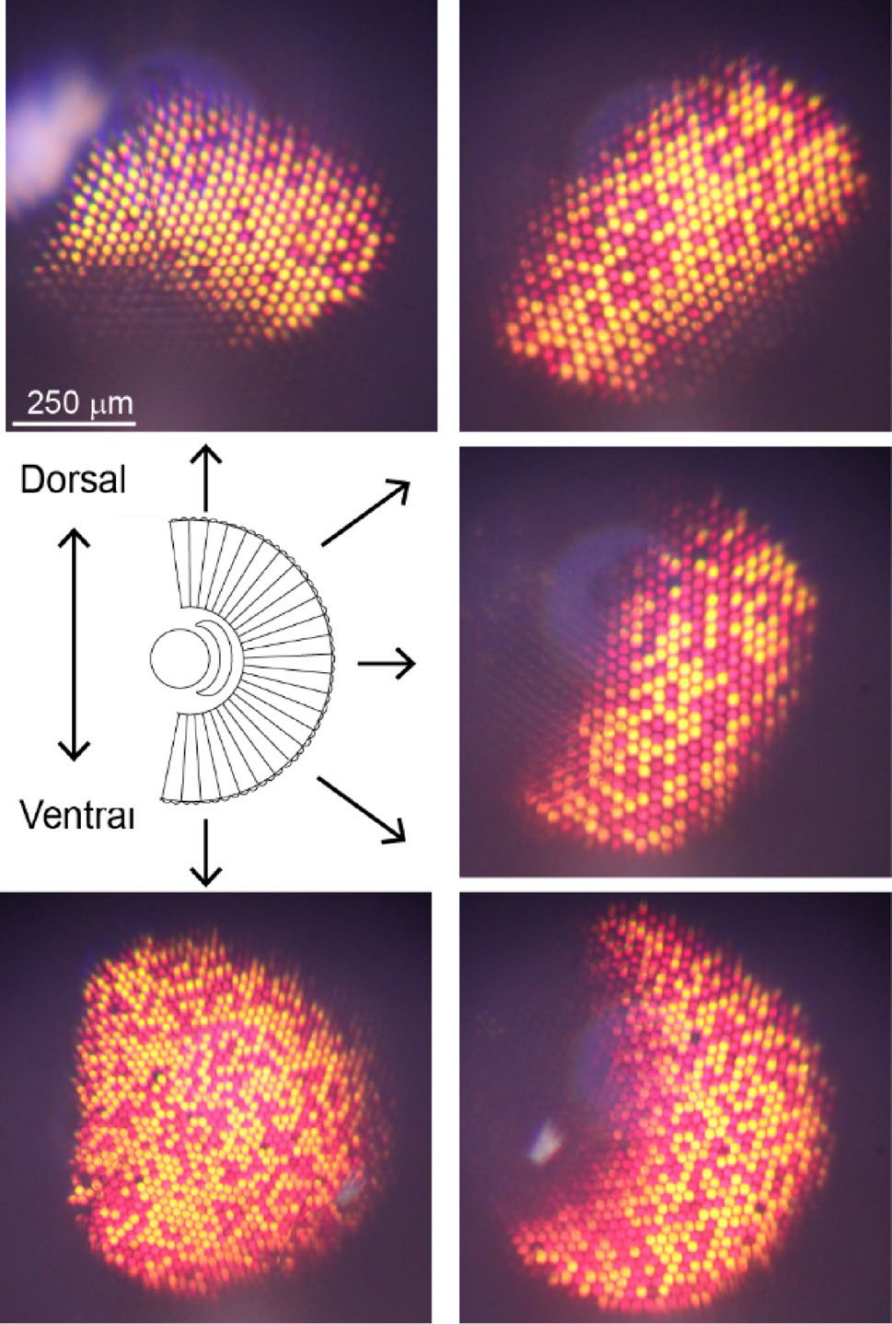
Example eyeshine images. The eyeshine was imaged across the entire dorsal-ventral axis of each butterfly eye. Shown here are a subset of the images for a single *H. pachinus* male. A red eyeshine is indicative that the ommatidium expresses a red screening pigment. The increased number visible in the ventral eye is due to a lower curvature of the eye that increases spatial resolution of the eye

## Results

**Fig. 3:**
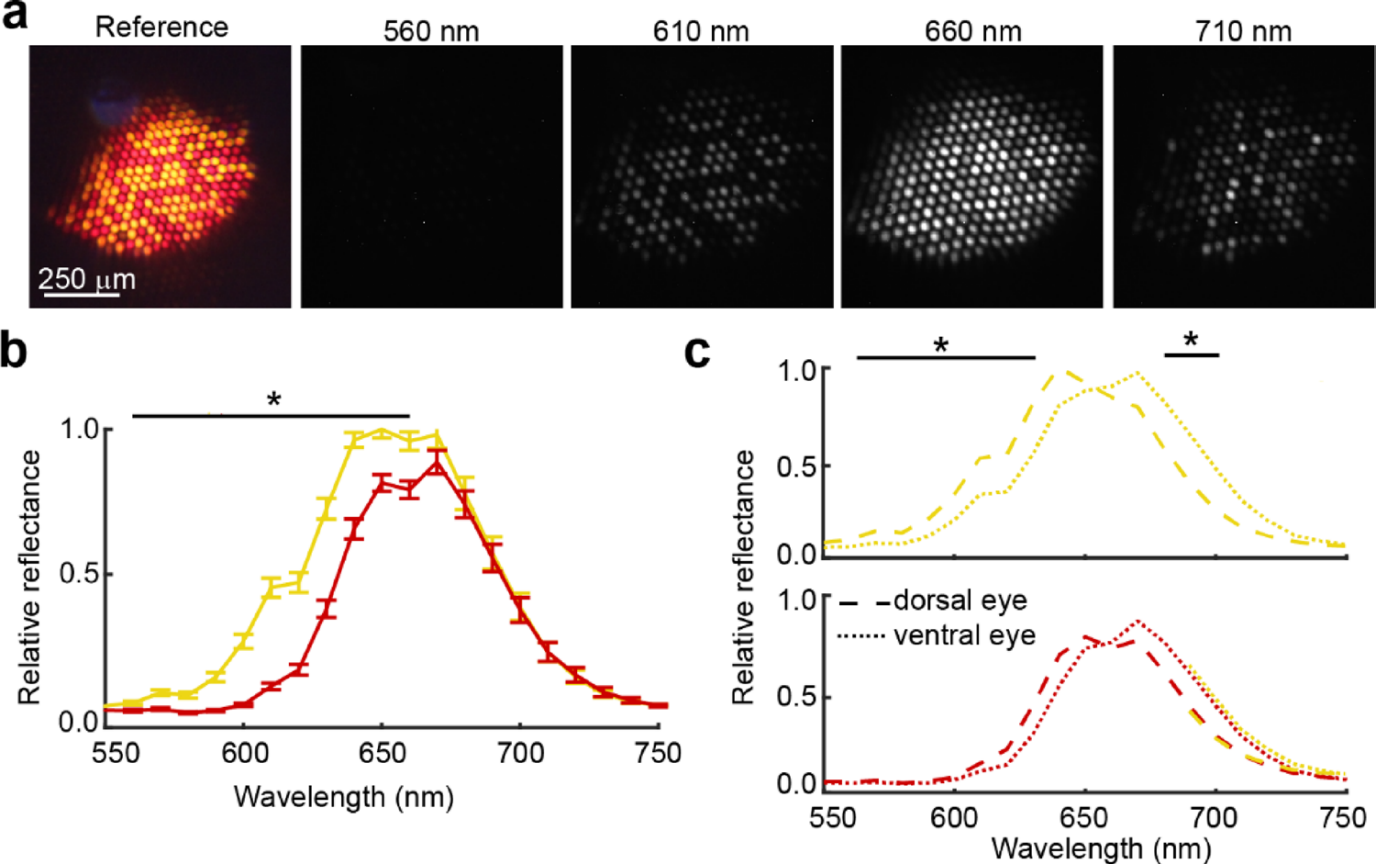
Eyeshine reflectance spectra. **a** Reflectance spectra for individual ommatidia were measured using a monochromatic camera and a series of monochromatic stimuli. Shown here are images from the middle of the eye in an *H. c. galanthus* male. **b** Average reflectance spectrum for red and yellow eyeshine (*n* = 24), grouped across species and sex because they were not significantly different (*p* = 0.85). Line color corresponds to eyeshine color. Asterisk indicates where red and yellow ommatidia had significantly different reflectance (*p* < 0.05 with Holm-Bonferroni correction). Error bars show mean ± SEM. **c** Red and yellow ommatidia were separated into the dorsal, middle, and ventral part of the eye. Line color corresponds to eyeshine color. Asterisks indicate wavelengths where the reflectance differs significantly across all three regions of the eye (*p* < 0.05, ANOVA with Holm-Bonferroni correction). The middle eye was intermediate to the dorsal and ventral. It and error bars are not shown for clarity. Long wavelengths for yellow ommatidia are copied onto the bottom panel to show similar shifts in tapetum reflectance for both eyeshine colors

**Fig. 4:**
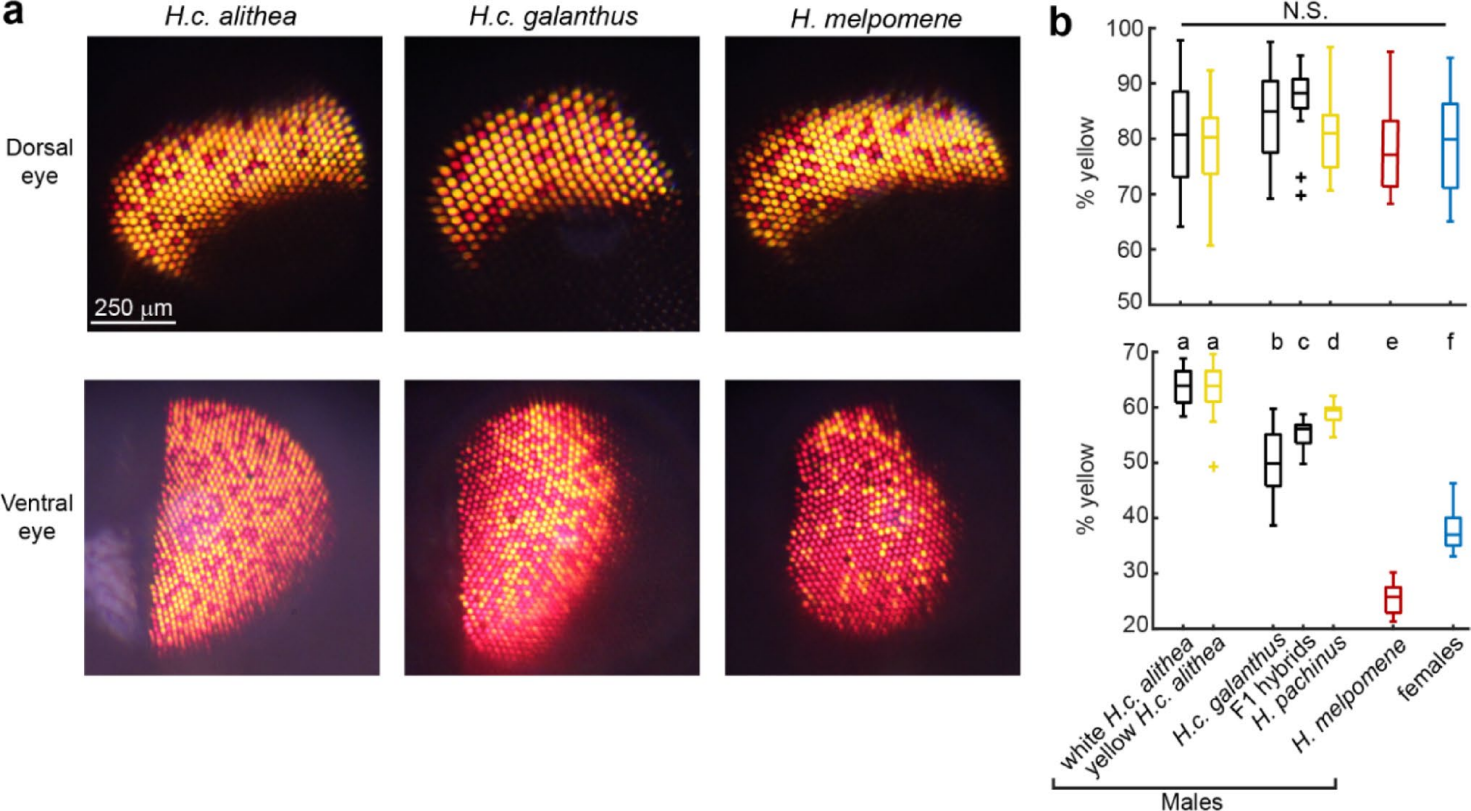
Distribution of eyeshine across eye.** a** Representative images from males show the distribution of red and yellow ommatidia at the most dorsal and ventral parts of the eye. **b** Boxplots show the proportion of yellow ommatidia in the dorsal (top) and ventral (bottom) eye. Groups with different letters are significantly different from each other (t-tests, *p* < 0.05 with Holm-Bonferroni correction, *n* = 15, 15, 15, 14, 14, 15, 15)

To characterize the organization of the eye across a closely related clade of H. cydno butterflies, we first used an epifluorescence microscope to measure the eyeshine (Fig. 2). This method gave us a coarse but global means to assess potential differences in eye organization, including the distribution of the basic and expanded Nymphalid mosaic (Ilić et al. 2022; Pirih et al. 2022). In agreement with eyeshine in other *Heliconius* species, every butterfly had some red ommatidia indicative of the expanded mosaic and some yellow ommatidia indicative of no pigment and the basic mosaic (Figs. 2, 3 and 4). Images from the ventral part of the eye had nearly twice as many ommatidia than the dorsal eye (476.8 ± 124.8 vs. 242.5 ± 37.7, *p* < 0.001) due to the increased spatial resolution.

Using a monochromatic camera paired with a series of monochromatic stimuli, we compared the reflectance spectra of red and yellow ommatidia in the dorsal and ventral eye (Fig. 3a). Yellow ommatidia had significantly greater reflectance than red for wavelengths between 550 and 660 nm along with a peak reflectance that was 17.2 ± 13.1% greater (t-test, *p* < 0.001) (Fig. 3b). The long wavelength cutoff is associated with filtering by the tapetum (Ribi 1979) and did not differ between the red and yellow ommatidia, with reflectance nearly absent above 730 nm. Ommatidia reflectance did not vary with species, sex, or wing color, but it did vary across the dorsal-ventral axis of the eye (Fig. 3c). Moving from dorsal to ventral, yellow ommatidia reflectance progressively shifted towards longer wavelengths. A similar but smaller and not significant shift was observed in the red ommatidia (*p* = 0.224). The main contributor for this dorsal-ventral shift is likely that ommatidium length varies from dorsal to ventral (McCulloch et al. 2016), resulting in a longer optical path in the ventral eye that allows opsins to absorb more light and red-shift the measured eyeshine. With no change in the shape of the reflectance spectrum or the peak intensity, we cannot fully rule out additional contributing factors.

Comparing the proportion of red and yellow ommatidia showed dorsal-ventral differences along with a sexual dimorphism where male eyes vary with species and female eyes do not (Fig. 4). The dorsal eye was predominantly yellow, and this did not vary across species (F_4,101_ = 1.16, *p* = 0.33) or sex (F_1,101_ = 3.62, *p* = 0.06) (Fig. 4a). In contrast, the proportion of yellow ommatidia in the ventral half of the eye varied significantly with species (F_4,102_ = 41.76, *p* < 0.001), sex (F_1,102_ = 127.01, *p* < 0.001), and there was a significant species X sex interaction (F_4,102_ = 37.8, *p* < 0.001). For every species, female eyeshine proportions were significantly different from males (*p* < 0.001, t-tests with Holm-Bonferroni correction). Furthermore, female proportions did not vary across species (F_5,14_ = 0.41, *p* = 0.83), with 37.9 ± 3.6% of ventral ommatidia having a yellow eyeshine. For males, all taxa had significantly different ventral eyeshine (*p* < 0.05 with Holm-Bonferroni correction). H. c. alithea had the highest proportion of yellow ommatidia (63.5 ± 4.2%), and there was no difference between individuals with white or yellow wings (*p* = 0.90). F1 hybrid offspring between H. c. galanthus (50.5 ± 6.1%) and H. pachinus (58.9 ± 1.9%) had an intermediate phenotype compared to the parental species (55.2 ± 2.5%). Compared to these H. cydno clade butterflies, the closely related H. melpomene had mostly red ommatidia in the ventral eye (yellow = 25.5 ± 2.9%).

**Fig. 5:**
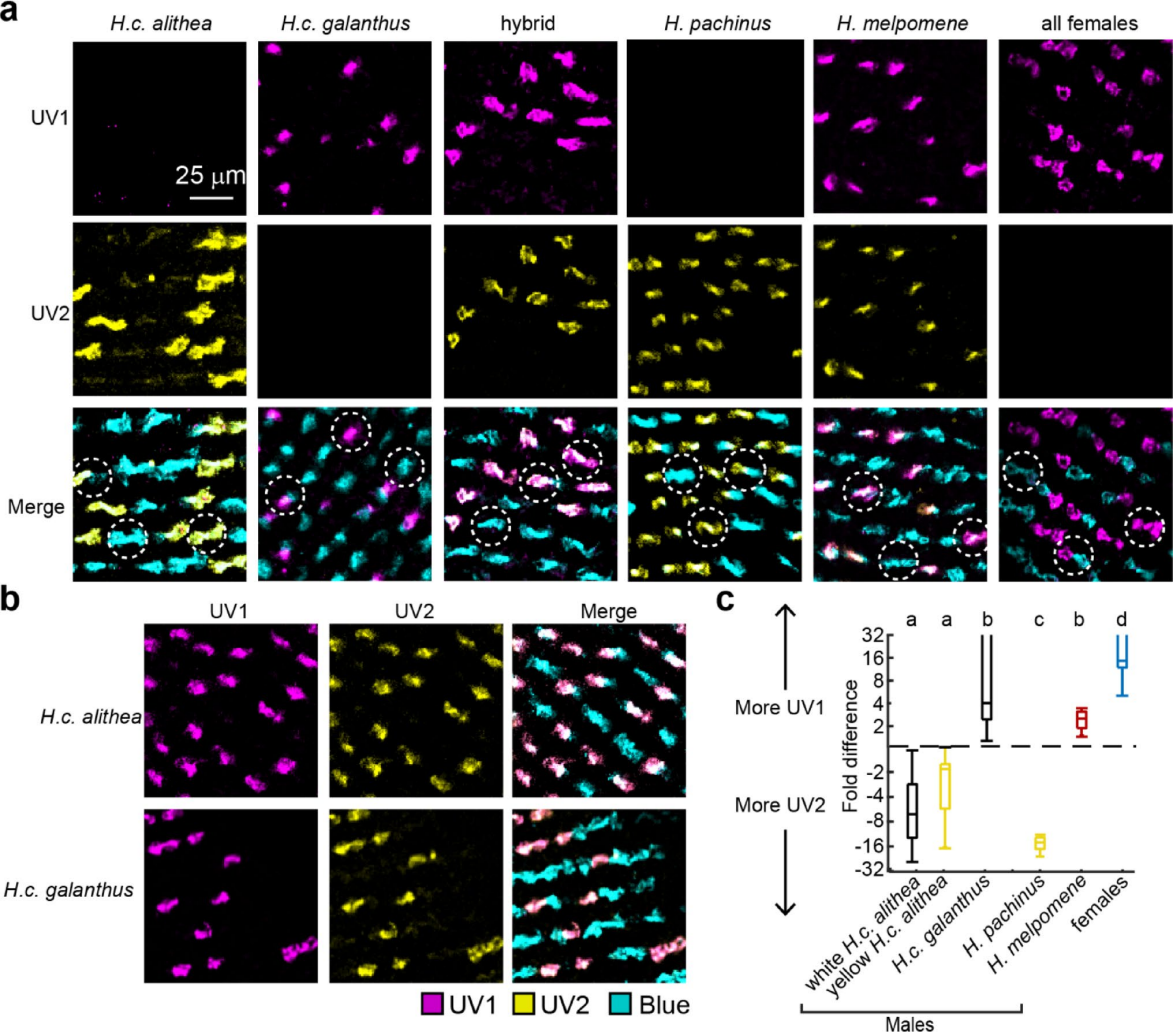
Variable co-expression of UV1 and UV2 opsins.** a** Antibody staining for UV1, UV2, and B opsins in thin cross sections of the eye. The female shown is a white *H. c. alithea* that is representative of all females (*n* = 14, 2–4 per species). In the bottom row, examples of UV-UV, B-B, and UV-B ommatidia are circled. **b** Representative antibody stains for *H. c. alithea* (*n* = 12 of 19) and *H. c. galanthus* (*n* = 3 of 14) that showed evidence of UV1 and UV2 co-expression. **c** Boxplot shows the relative expression levels of UV1 and UV2 on a log scale using qPCR. No UV2 was detected in some females and *H. c. galanthus* males. Each box plot includes 12 individuals, but no F1 hybrids were used for this experiment. Letters above each box indicate groups that are significantly different from each other (t-tests, *p* < 0.05 with Holm-Bonferroni correction)

Observing these differences in eyeshine across species and sex, we then turned to immunohistochemistry to identify the distribution of UV1, UV2, and blue opsins across the eye. Consistent with a previous report, every butterfly had a combination of UV-UV, B-B, and UV-B ommatidia, defined by expression in R1 and R2 photoreceptors (Fig. 5) (McCulloch et al. 2017). More recently, co-expression of B and LW opsins in a subset of R1/R2 photoreceptors was reported across several *Heliconius* species, for a total of 6 ommatidial classes in the closely related *H. melpomene* (McCulloch et al. 2022). Electrophysiology from *H. cydno* butterflies suggests a similar organization (VanKuren et al. 2025), but our immunohistochemistry here did not use an LW antibody to confirm this expression. Matching the eyeshine results and previously reported electrophysiology, opsin immunohistochemistry again showed a sexual dimorphism where males eyes vary with species and female eyes do not. Although the ommatidial classes present in each butterfly did not vary, the specific UV opsin expressed in a UV photoreceptor did (Fig. 5a). All females (*n* = 14) regardless of species identity expressed only UV1. In *H. pachinus* males (*n* = 4) we detected only UV2, while its sister *H. c. galanthus* (*n* = 14) predominantly expressed UV1, although UV2 co-expression was detected in three males (Fig. 5b). F1 hybrid offspring of these two species (*n* = 11) had an intermediate phenotype where every male strongly co-expressed UV1 and UV2. Every *H. c. alithea* male (*n* = 19) expressed UV2 but also expressed large variability in UV1 expression where UV1 expression was strong in 4 males, weak in 8 males, and absent in 7 males (Fig. 5b). Strong co-expression was also observed in every H. melpomene male (*n* = 13).

Using antibody staining to assess the degree of UV1 and UV2 co-expression is limited to qualitatively scoring expression as strong, weak, or absent. To further quantify UV1 and UV2 co-expression both within and across species groups, we turned to performing qPCR for UV1 and UV2 transcripts in whole eyes (Fig. 5c). Transcripts for both opsins were detected in most but not all eyes, although often at very low levels. For example, H. pachinus males consistently had UV2 expression levels 16X greater than UV1. Consistent with antibody staining, we also observed a huge variability within H. c. alithea, where some males had equal levels of UV1 and UV2, while others could have UV2 25X times greater than UV1. Overall, both the mean ratios and the within species variability were consistent with what we observed using antibody staining.

**Fig. 6:**
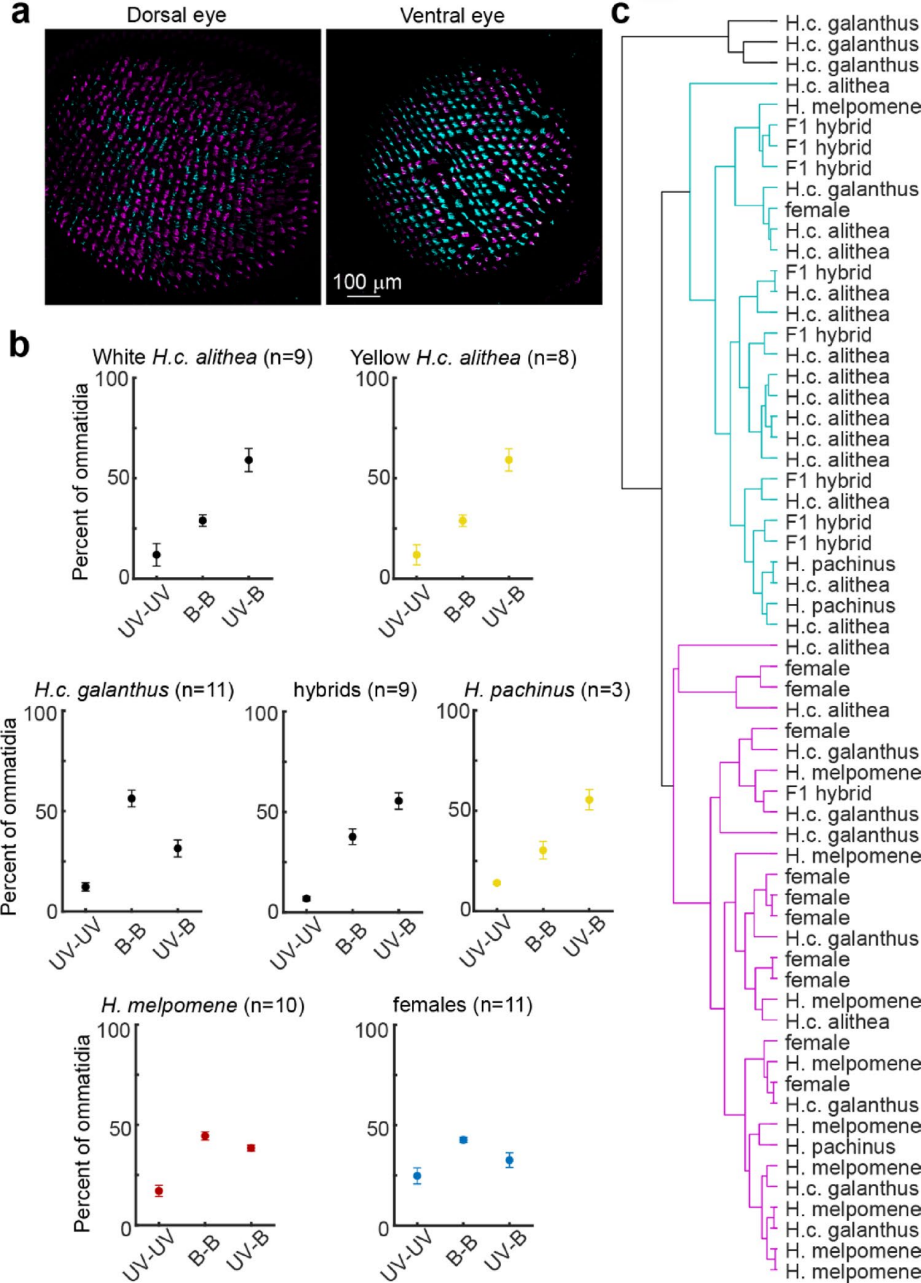
Distribution of three ommatidial classes across the eye.** a** Example antibody stains comparing the dorsal eye that is predominantly UV photoreceptors and the ventral eye that is a more equal mix of UV and B. **b** The proportion of each ommatidial class was measured for ventral eye. Panels labeled with species names are males only, while females are split across all groups H. pachinus and F1 hybrids. Plots show mean ± SEM **c** To compare distributions across groups, we used hierarchical clustering of the data in panel b. Two major clusters indicated by colors are largely delineated by group identity

Finally, we wanted to ask if the distribution of these three ommatidial classes varied across the eye or between species (Fig. 6). We first observed a clear dorsal-ventral difference, where UV-UV ommatidia dominated the dorsal eye while the ventral eye was more equal (Fig. 6a). We used hierarchical clustering to determine if these ventral eye distributions varied across groups and identified two major clusters that segregated strongly based on species and sex. (Fig. 6b-c). One cluster primarily included *H. c. galanthus* males, *H. melpomene* males, and all females. These eyes were distinguished by a large fraction of B-B ommatidia (~ 50%) and a small fraction of UV-UV ommatidia. A minor cluster distinct from this one included three *H. c. galanthus* males with an especially low proportion of UV-UV (< 5%). The second major cluster included *H. c. alithea* males, *H. pachinus* males, and F1 hybrid offspring males. For these eyes, the proportion of UV-UV was also small, but UV-B was the primary ommatidial class (~ 60%).Within these two clusters, there was no additional clustering based on species, suggesting a similar organization across species within each cluster.

## Discussion

Sexually dimorphic eye organizations are commonly observed across butterflies (Arikawa et al. 2005; Sison-Mangus et al. 2006; Ogawa et al. 2012; Ilić et al. 2022), including species within *Heliconius* (McCulloch et al. 2017). Most prior comparisons, both within and outside *Heliconius*, have primarily focused on identifying the distinct ommatidial classes across species representative of different phylogenetic clades. We took a complementary approach to assess diversity among a set of closely related taxa where we identified sex-limited diversity such that male eyes vary and female eyes do not. This sexual dimorphism extended beyond the *H. cydno* clade as female *H. melpomene* did not differ from other females. Observing differences in screening pigment expression, which UV opsin is expressed, and the distribution of the measured ommatidial classes across the eye builds on previous *H. cydno* work where we similarly showed that UV photoreceptor electrophysiology also varies only for males (VanKuren et al. 2025). Whether this sex-limited diversity is a common theme in butterflies or specific to *H. cydno* is an intriguing question that will require future studies on other closely related species.

Eye organization diversity in *Heliconius* is largely defined by the expression patterns of UV1 and UV2, with at least six retinal mosaics described (McCulloch et al. 2017). Mapping these patterns onto the phylogeny shows that this diversification largely occurred near the base of this adaptive radiation of *Heliconius* butterflies. Previous work suggested that the melpomene/cydno clade had lost UV2 expression (McCulloch et al. 2017, 2022), in contrast to the results reported here (Fig. 5). There are two potential explanations for this difference. First, this difference in UV2 opsin detection could suggest natural biological variation within species. This explanation would be consistent with the within-species variability we observed for *H. c. galanthus* and *H. c. alithea*. Alternatively, this difference might suggest differences in the binding affinity of the different antibodies used in the two studies. Prior RNA-Seq in *H. melpomene* (McCulloch et al. 2017) agrees with our qPCR results showing an approximately 3X ratio of UV1 to UV2 for *H. melpomene* males. Co-expression is further supported by physiology in *H. melpomene* (McCulloch et al. 2022) and*H. cydno* (VanKuren et al. 2025), where UV photoreceptor sensitivities are intermediate to the 355 and 390 nm expected for UV1 and UV2, respectively. This continuous variability in spectral tuning can best be described by the co-expression we observed. We also did not detect UV2 in any female, consistent with qPCR and supporting the specificity of the antibody. Together, these results might suggest the new UV2 antibody developed for this study can detect lower expression levels that do influence photoreceptor physiology.

*Heliconius* can express UV1 and UV2 in separate photoreceptors (McCulloch et al. 2017), so the functional role of co-expression remains unclear. In *H. erato* and other species, female expression of UV1 and UV2 in different photoreceptors functionally expands the range of color vision into UV compared to butterflies with only one UV opsin (Finkbeiner and Briscoe 2021). Males instead express only UV2, which functions to improve conspecific identification compared to UV1 by enhancing discriminability of a genus-specific yellow pigment used for wing coloration (Briscoe et al. 2010; Bybee et al. 2012). One possibility is that fine control over the specific wavelength of peak sensitivity confers some selective advantage. A subset of *H. doris* photoreceptors co-express UV1 and UV2, supporting this idea (McCulloch et al. 2017). Alternatively, the original loss of separate photoreceptors may impose a developmental constraint, and co-expression might balance the adaptive value of UV1 and UV2. Finally, chromatin organization appears to determine which UV opsin is expressed in *H. melpomene* (McCulloch et al. 2022), so the low levels of co-expression observed could instead be due to incomplete suppression from this mechanism.

The biggest difference we observed across taxa was the distribution of red screening pigment in the ventral eye. Yellow eyeshine corresponds to the basic Nymphalid retina, while red eyeshine corresponds to the expanded retinal mosaic and the presence of green vs. red opponency (Fig. 1) (Ilić et al. 2022; Pirih et al. 2022). In the *Heliconius* expanded mosaic the LW expressing R1/2 cells co-express blue (McCulloch et al. 2022), indicating that our stains would still mark most or all ommatidia. This is consistent with our visual inspection of the staining showing few gaps in ommatidia tiling of the slices. Assuming an equal probability of any B cell co-expressing LW, a simple least squares approach relating the proportion of red eyeshine to the proportions of ommatidial classes suggests 70–80% of B cells in females and *H. melpomene* males co-express LW, while only 30–40% co-express in *H. cydno* males (see methods for details). While these values will require future validation using an LW opsin, this large shift towards less LW expression in R1/R2 cells is consistent with the large shift towards yellow eyeshine in *H. cydno* males and could present a useful system for exploring the genetics, development, and behavioral impact of the expanded mosaic. Eyeshine data from other *Heliconius* species is limited, but *H. erato* also exhibits a high proportion of red eyeshine (McCulloch et al. 2016), raising the question of whether this shift towards the basic mosaic is unique to *H. cydno* males and the degree of variability across the genus.

We also observed differences in the overall distribution of UV and blue expressing photoreceptors. Females and *H. melpomene* males clustered together with predominately B-B ommatidia, potentially showing the ancestral distribution. *H. c. galanthus* males were similarly included in this cluster, which is interesting because yellow wings are the ancestral state of *H. cydno*. The white wings of *H. c. galanthus* are derived, which could suggest a secondary reversion to this distribution. The F1 hybrids were similar to and clustered with *H. pachinus* and *H. c. alithea*, further suggesting that the distribution with predominately UV-B ommatidia could be the dominant phenotype. Individuals within each cluster did not further segregate based on species identity even though we did observe eyeshine differences. This is most apparent with *H. c. galanthus* having more than double the proportion of yellow eyeshine compared to *H. melpomene* and could suggest that opsin expression and the development of the basic vs. expanded mosaic are largely under independent control.

Butterflies live in environments with variable ambient light conditions where they rely on their visual systems for foraging, courtship, and oviposition. Every species studied here lay eggs on *Passiflora* vines (Smiley 1978), so the lack of variability could point towards an eye adapted for host plant identification. Red sensitivity is important for oviposition in the distantly related *Papilio aegeus* (Kelber 1999), and the large proportion of red eyeshine corresponding to the expanded mosaic could serve a similar function here. With red wings, *H. melpomene* may similarly benefit from the strong bias towards red eyeshine for conspecific identification. *H. c. alithea*, *H. pachinus*, and the hybrids will all court yellow females, so the similar opsin distributions with mostly UV-B ommatidia as well as the primarily UV2 expression could also function as adaptations for mate detection. *H. cydno* also lives in closed forests while *H. melpomene* prefers brighter forest edges (Estrada and Jiggins 2002). This difference places selective pressures on eye morphology (Wright et al. 2024), and eye organization differences observed here could function as additional adaptations. Together, our results show that different features of retinal organization can vary substantially among closely related taxa and highlights the evolvability of the peripheral nervous system.

## Data Availability

All of the data presented here are available from the Dryad Digital Repository at 10.5061/dryad.5hqbzkh7v.
